# Nursing care supervision: A qualitative study of staff and auxiliary nurses’ experiences in Limpopo province

**DOI:** 10.4102/hsag.v31i0.3251

**Published:** 2026-07-27

**Authors:** Munyadziwa R. Raliphaswa, Takalani R. Luhalima, Julia L. Mafumo, Vhonani O. Netshandama

**Affiliations:** 1 Department of Advanced Nursing Science, Faculty of Health Sciences, University of Venda, Thohoyandou, South Africa; 2 Directorate of Community Engagement, Entrepreneurship, Inclusive Innovation Commercialisation, University of Venda, Thohoyandou, South Africa

**Keywords:** experiences, group research, Limpopo College of Nursing, student nurse, supervisors

## Abstract

**Background:**

According to the South African Nursing Council’s regulation R 2127, staff and auxiliary nurses must be supervised by professional nurses. This supervision is essential for quality care, leading to fewer patient complaints and higher satisfaction.

**Aim:**

This study aimed to explore and describe the experiences of staff nurses and auxiliary nurses on the supervision of nursing care by professional nurses in Limpopo province.

**Setting:**

The study was conducted in three selected regional hospitals located in different districts of Limpopo province: Vhembe, Mopane, and Waterberg.

**Methods:**

A qualitative approach using exploratory, descriptive, and appreciative inquiry research designs was selected to gain insights into professional nurses’ supervision of nursing care. Non-probability purposive sampling was used to choose districts, hospitals, units, and participants. Data were collected through focus group interviews with participants from paediatric, maternity, and casualty units and analysed using Tesch’s eight steps of data analysis.

**Results:**

Four themes emerged: Staff shortages, educational and training challenges, attributes of a good supervisor, and the roles and responsibilities of supervisors.

**Conclusion:**

To improve the quality of patient care outcomes and reduce patient complaints and lawsuits, professional nurses must supervise nursing care. However, the current difficulties jeopardise professional nurses’ ability to optimally supervise nursing care.

**Contribution:**

This study contributes to improving the quality of supervision of nursing care, which is aimed at achieving high patient satisfaction, reducing patient complaints and related medico-legal negligence cases. This study also demonstrates how effective supervision by registered professional nurses can enhance the knowledge and skills of both staff nurses and auxiliary nurses.

## Introduction

Nursing is the foundation of health care because admitted patients are cared for by nursing staff around the clock. To provide high-quality nursing care, professional nurses must continuously supervise staff nurses and auxiliary nurses. According to the South African Nursing Council’s (SANC) position statement (SANC 2021), supervision is a professional activity in which less experienced nurses benefit from their supervisors’ knowledge and experience to address knowledge or skill gaps.

The Regulation for the Scope of Practice of Nurses and Midwives (R2127) further states that the staff nurses and auxiliary nurses must work under the supervision of the professional nurses (SANC 2022).

Supervision is an important strategy that complements the quality of nursing practice in implementing actions to improve patient satisfaction and ensure the quality of nursing care. Therefore, support and daily monitoring of patient care define the path for staff nurses and auxiliary nurses to gain knowledge and skills (Brás Baptista Sérgio, Rodrigues Faria de Carvalho & Correia Barroso Pinto 2023). The SANC emphasises the importance of supervision in nursing care, stating that it fosters professional development and improves performance (SANC 2022). Therefore, staff nurses and auxiliary nurses need to grow professionally so that nursing care can be of high quality (SANC 2021).

Supervision of nursing care is necessary to improve patient care, support professional development, and maintain ethical standards in the nursing profession. Nonetheless, it has been found that difficulties, including a heavy workload and a demanding workplace, make it difficult for professional nurses to supervise auxiliary nurses and staff nurses. Moreover, the lack of effective supervision of nursing care contributes to compromised patient safety and medico-legal negligence cases (Shongwe, Downing & Nene 2024).

The effective execution of supervision requires a conducive therapeutic environment between nursing staff and patients, despite the challenges that can arise, which can result in ineffective supervision of nursing care (Achempim-Ansong, Kwashie & Ofei 2021). Effective supervision produces competent nurses who can render quality nursing care. Even so, many professional nurses may not have been adequately trained to supervise nursing care when they first started working; therefore, their ability to do so may vary (Mabusela & Ramukumba 2021). The consequences of ineffective supervision are evident, with numerous cases of substandard care, patient negligence and legal disputes arising. As of February 2022, medico-legal claims against the Limpopo Health Department surpassed R14 billion, largely attributed to negligence cases in the Vhembe district (Limpopo 2022).

Supervision of nursing care has been implemented globally, locally, and in African countries for a long time; however, despite this, some challenges hinder its effective implementation. Globally, nursing care supervision is regarded as essential for enhancing nurses’ professional development and improving healthcare quality through guidance and mentorship. High-income countries, including the United Kingdom, Australia, and Canada, have implemented formal frameworks for professional development and supervision (Martina et al. 2026). Despite its recognised benefits, a study in Victoria, Australia, by Ryu et al. (2025) identified barriers such as organisational culture, staff shortages, and workload that impede effective supervision. Conversely, another Australian study by McDonough, Rhodes and Procter (2025) found that supervision enhances workers’ skills, knowledge, and job satisfaction.

Effective supervision of nursing care in Africa faces challenges such as high patient-to-nurse ratios, staff shortages, inadequate supervisory skills, and task pressure (Alhassan et al. 2025). A study about supervision in sub-Saharan countries by Serapelwane, Bam and Kovane (2023) indicates that effective supervision is crucial for enhancing health systems in sub-Saharan nations, improving performance and health service delivery. However, supervision is often hindered by resource scarcity, excessive workloads, understaffing, and inadequate supervisor training, leading to a focus on evaluation rather than professional development.

In South Africa, nursing care monitoring aims to improve healthcare quality despite challenges like staffing shortages and financial constraints. Professional nurses play a vital role in overseeing care, ensuring compliance with standards, and enhancing patient safety (Shongwe et al. 2024). A study in Gauteng by Shongwe et al. (2024) highlights that supervision improves task management and clinical judgement among nurses. However, effective nursing supervision in public healthcare is hindered by issues such as high patient-to-nurse ratios, heavy workloads, a lack of managerial support, and limited resources.

The aim of this study was to explore and describe the experiences of staff nurses and auxiliary nurses regarding the supervision of nursing care by professional nurses in the selected regional hospitals of Limpopo province.

Proctor’s integrated supervisory model serves as the theoretical backdrop for the study, which aims to enhance outcomes for users and clinicians by supporting nursing supervision (Snowdon et al. 2020). Oktavia et al. (2023) emphasise the need for effective supervision to enable healthcare workers to accomplish their tasks. The model comprises three domains: Formative, normative, and restorative, and is recognised for promoting professional growth, compliance with clinical standards, and the development of knowledge and skills within the nursing field.

Normative supervision establishes ethical standards for supervisees, restorative supervision fosters their development, and formative supervision enhances their skills. Proctor’s model highlights the formative component, enabling nurses to improve their knowledge and practice, which positively influences nursing care supervision. Normative supervision enforces adherence to nursing standards among staff, thereby promoting quality and clinical audits (Litherland et al. 2023).

## Research methods and design

### Research design

This study employed a qualitative approach using the exploratory, descriptive, and appreciative inquiry research design. This approach was used to explore and describe the experiences of staff nurses and auxiliary nurses regarding supervision of nursing care by professional nurses in the selected regional hospitals of Limpopo province (Polit & Beck 2021). The appreciative inquiry design was also chosen because the researcher wanted to identify what is working well and what is not in an organisation regarding the supervision of nursing care by professional nurses (Armstrong, Holmes & Henning 2020).

### Research setting

The study was conducted in Limpopo province, South Africa, which has a population of 6.5 million (Stats SA 2020). Limpopo province has five district municipalities, namely Vhembe, Waterberg, Capricorn, Mopani, and Sekhukhune. To serve its large population, there are 43 hospitals, 471 clinics, and 22 health centres. The study was conducted in three districts: Vhembe, Mopane, and Waterberg. These districts were chosen because of the number of medico-legal negligence cases reported. The Vhembe district has the highest number of medico-legal negligence cases, with 500 amounting to R5.2 bn; and the Mopani district has 322 amounting to R2.6 bn; whereas the Waterberg district has 119 amounting to R766 mn (Limpopo DA 2022). The three regional hospitals were selected for this study because they serve as the district hospitals’ referral centres.

### Population and sampling

A total of 37 nurses participated in the study, comprising 18 staff nurses and 19 auxiliary nurses. Six focus groups were conducted, each with six participants, except the last, which had seven. Participants were selected as they could provide detailed information about their experiences with the supervision of nursing care by professional nurses within the selected hospitals.

A non-probability purposive sampling method was used in this study, whereby participants were selected based on the researcher’s judgement and their in-depth knowledge of the supervision of nursing care by professional nurses (Brink & Van Rensburg 2022). The purposively selected units were Paediatric, Maternity, and Casualty because the patients managed were of high acuity; therefore, the supervision of nursing care should be of high quality to prevent loss of lives and patient complaints. Participants selected had at least 6 months of clinical experience in the sampled units, ensuring sufficient exposure to supervision by professional nurses. Written informed consent was obtained from all participants. The sample size was determined by data saturation, even though data saturation was reached at focus group number four. Still, the researcher continued with data collection with two more focus groups to authenticate the findings (Brink & Van Rensburg 2022).

### Data collection

Since individuals are best understood in their living settings, data were collected in the Maternity unit, Paediatric ward, and Casualty department of the selected regional hospitals where the participants work.

To learn more about the experiences of staff nurses and auxiliary nurses regarding nursing care supervision by professional nurses, the researcher conducted focus group interviews using interview guides (Gray, Grove & Sutherland 2017). Appreciative inquiry design served as the foundation for the interview guide. The researcher used appreciative inquiry questions to initiate transformative discussions with nurses in the selected regional hospitals. The nursing manager recruited the participants. The managers had already set up the location, which included the boardrooms and dining rooms at the chosen regional hospitals. Informed consent was obtained when the participants were informed about the study by the researcher. Focus group interviews were conducted with the researcher as the facilitator. Data were collected between May 2024 and September 2024 over 12 days.

The questions asked were: (1) What are your experiences regarding supervision of nursing care by professional nurses in your hospital? (2) What do you think needs to be improved when it comes to the supervision of nursing care by professional nurses in your hospital? (3) How best do you think professional nurses should supervise the nursing care in your ward and hospital? (4) In the coming years, what changes would you like to see introduced in your hospital to improve supervision of nursing care by professional nurses?

These questions were developed based on the four dimensions of Appreciative Inquiry: discovery, dream, design, and destiny. Participants were encouraged to openly discuss their experiences regarding the supervision of nursing care by professional nurses. A voice recorder was used with their permission; the researcher employed probing to ensure all topics were covered, and the researcher took notes during interviews to document their informal behaviour (Polit & Beck 2021). Each focus group interview took 60 min – 90 min.

### Data analysis

The researcher used Tesch’s eight steps to analyse the data as mentioned in Creswell and Creswell (2023). Data from focus groups and field notes were transcribed verbatim. Data were meticulously inspected and analysed accordingly. Data highlighted the real experiences of staff nurses and auxiliary nurses during the supervision of nursing care by professional nurses. The written data from the transcripts were read several times to capture similar information about the experiences of staff nurses and auxiliary nurses regarding the supervision of nursing care. The data were categorised in a meaningful and organised way, with similar topics grouped together. Assembled data belonging to each category in a place were analysed and coded as themes. The supervisor assisted with the coding of the findings.

The researcher compiled the themes and sub-themes into a summary. After sending the raw data to a skilled independent coder, a consensus was reached on the identified themes and sub-themes.

### Trustworthiness

According to Polit and Beck (2021), Lincoln and Guba’s model of trustworthiness includes dependability, confirmability, credibility, and transferability to guarantee trustworthiness. The study ensured that measures to ensure trustworthiness were observed and maintained. Credibility was ensured through multiple sources, including combining focus group interviews with field notes and participant observation, and reviewing findings and summaries with participants to ensure the analysis reflects their opinions. Data was collected through audio-recorded focus group interviews and field notes (Fouche, Strydom & Roestenburg 2021).

Transferability was enhanced by purposive selection of districts, hospitals, units, and participants.

Additionally, the researcher thoroughly described the research setting, study participants, and research process (Grove & Gray 2022). Confirmability was enhanced by using an audio recorder to capture participants’ responses in their original, unaltered state. Confirmability was further ensured through systematic data analysis, using direct participant quotes to maintain authenticity and accurately reflect their perspectives (Polit & Beck 2021). To improve dependability, an audit trail of the research procedures was maintained, including a thorough explanation of the methods and transcripts, which were provided to an independent coder for analysis. Dependability was maintained through an in-depth description of the research methodology used, with all steps clearly stated. The researcher remained focused on the research problem and the study’s objectives. This independent researcher examined the documentation of the critical incidents and the investigation process, including data, findings, interpretation, and recommendations (Polit & Beck 2021).

### Ethical considerations

Ethical clearance to conduct this study was obtained from the University of Venda Higher Degree Ethics Committee under reference number FHS/23/PDC/01/1409 in September 2023. Approval was also sought from the Limpopo Department of Health Ethics Committee, as well as from the three district health offices and three selected regional hospitals in Limpopo province. The researcher ensured that ethical principles were upheld by protecting participants’ privacy and confidentiality throughout the study. Consent to participate was obtained from each participant voluntarily, without coercion, and they had the right to withdraw at any time without penalty. Participants’ identities were kept confidential, with no personal names or identifying information recorded. Instead, code names such as FG 1: M1 were used. All digital data was secured with passwords, while hard copies were stored in a locked cupboard.

## Results

### Participant’s demographic data

Thirty-seven staff nurses and auxiliary nurses participated in the study; of these, 31 were female, and 6 were male. There were few males because nursing is a female-dominated profession. The participants from Maternity were 13, from Casualty units were 12, and from the Paediatric ward were 12. The interviewed participants’ ages ranged from 40 to 60 years, and their years of experience ranged from 10 to 40 years. Data was collected through six focus group interviews for both categories of nurses. Each focus group had 2 staff nurses and 2 registered auxiliary nurses, for a total of 18, from the three wards and/or units of three selected regional hospitals.

However, 1 regional hospital had 3 auxiliary nurses, bringing the total to 19.

### Presentation of the findings

After reading and re-reading the transcripts, the researchers extracted 4 themes and 10 sub-themes: Staff shortages, education and training challenges, and the supervisor’s roles and responsibilities. These findings were underpinned by Proctor’s supervision model and were based on the experiences of participants as outlined in their own voices. The findings were grouped into themes and subthemes as shown in Table 1.

**TABLE 1:** Summary of themes and sub-themes.

Themes	Sub-themes
1. Shortage of staff	1.1. Increased workload 1.2. Absenteeism 1.3. Fatigue
2. Educational and training challenges	2.1. Lack of staff training 2.2. Lack of training of supervisors
3. Suggested attributes of a good supervisor	3.1. Approachable and avoid favouritism 3.2. Good communication skills
4. Supervisory role and responsibility of professional nurses	4.1. Delegation of staff 4.2. Direct supervision 4.3. Rewarding good performances

#### Theme 1: Shortage of staff

Data on the experience of being supervised by professional nurses was collected from staff nurses and auxiliary nurses. Subsequently, the participants informed the researcher about the difficulties they encountered when supervising nursing care, including staffing shortages, difficulties with education and training, features of effective supervisors, and the duties and responsibilities of supervisors.

Shortage of staff was cited by the majority of participants as a challenge in most hospitals and was due to unfilled posts after the exit of nurses, either due to death, retirement, or resignation from the management.

**Sub-theme 1.1: Increased workload:** This sub-theme explores the consequences of a staffing shortage as reported by participants. The department’s biggest problem, according to all research participants, is a lack of staff, which makes it difficult to oversee nursing care. The supervision of nursing care is hampered by a shortage of nursing staff in all categories, which is increasing workload, exhaustion, burnout, and absenteeism. All of these put nursing care supervision at risk. The following are the remarks from participants highlighting the increased workload resulting from the shortage of staff:

‘Supervision, to me, I can say it is difficult because of the shortage of staff, as one can find that there is only one auxiliary nurse and one registered enrolled nurse, together with one professional nurse who should attend to the whole ward routine. This makes it difficult for us to do all the tasks because there is an increased workload that makes us fatigued.’ (FG2, member 12, male participant)

‘It is evident to me that the other colleagues are correct when they say that supervision is challenging due to the busy wards, staffing shortage, and excessive workload for the few nurses who are on duty. The workload seems excessive to me; the department should definitely think about recruiting more employees, as this is having a detrimental effect on our lives.’ (FG 4, member 22, female participant)

**Sub-theme 1.2: Absenteeism:** Participants reported absenteeism as a significant obstacle to nursing care supervision, as it is a contributing factor to the staffing deficit. The more employees miss work, the greater the staffing shortfall, which adversely affects patient care and makes supervision of nursing care more difficult. The participants also reported that, most of the time, there are very few nursing personnel on duty, who are not able to render quality patient care. The following quotations from participants verify this finding:

‘The other challenge we are experiencing is the challenge of absenteeism, which results in a shortage of staff and affects the quality of care that can be rendered. The employer should try to address this challenge as it is negatively impacting nursing care.’ (FG5, member 30, female participant)

‘What I can say is that, the standard of supervision has gone down because, from the era that we are coming from, the issue of resources was allocated fairly within all wards, but now we are experiencing the challenges of a shortage of resources, which stems from the equipment, supplies, and medication. All these resources are very vital when it comes to managing patient care, but the unit is running without these vital resources.’ (FG6, member 37, male participant)

Absenteeism is a problem that many participants mentioned, and it affects nursing care supervision. According to several participants, the staff’s increased burden because of the personnel shortage also motivated them to miss work. The ongoing staffing deficit because of absenteeism, which is a problem for nursing care monitoring, has a detrimental effect on the quality of nursing care.

**Sub-theme 1.3: Fatigue:** Most of the employees stated that they are growing increasingly exhausted due to their staffing shortage; as a result, they are only meeting the needs. This made it difficult to supervise nursing care, which affected the quality of patient care. Participants reported that, from professional nurses to the lowest level of registered auxiliary nurses, the system is being affected by weariness. The issue of weariness was raised by the participants, who noted that it contributes to the staffing deficit by increasing the number of employees who miss work. The following are the quotes from the participants:

‘The nurses are fatigued by too much work they are faced with due to overworking, as the ward is full of patients, but very few staff. Eish, it is painful because we are always tired and there is no time to rest. We don’t have time for tea or lunch breaks.’ (FG2, member 12, female participant)

‘Eish, the other issue that is challenging is nurses’ fatigue and exhaustion; it is because of staff being overworked due to a shortage of staff, which makes them burn out. I strongly feel that it needs to be addressed to revive their morale.’ (FG3, member 18, female participant)

#### Theme 2: Education and training challenges

Another obstacle to efficient nursing care supervision, according to the participants, is a lack of education and training. Other participants indicated that the tactics required for staff development and capacity building are employee education and training, as their absence compromises nursing care supervision and work performance. Because the staff is underprepared, some participants stated that a lack of education and training is a contributing factor to poor work performance. The sub-themes that emerged were a lack of staff training and supervisor training.

**Sub-theme 2.1: Lack of staff training:** Nursing staff training is crucial because it facilitates the sharing of knowledge and skills, particularly in the areas of medicine and clinical care. Most participants stated that exhaustion and a lack of enthusiasm for their work were caused in part by inadequate training. They believed they had lost their inspiration and had grown uninspired. They stated that they have not received any more training since their most recent training in 2019. They added that they are not even receiving in-service training to help them become more capable. The following are the direct quotes from participants:

‘Staff nurses and auxiliary nurses, we really want the training to come back so that we can be trained and progress from one level to the other. But because of a lack of training, we find ourselves being stagnant and also lacking knowledge of some of the procedures that are within our scope.’ (FG2, member 11, female participant)

‘All the hospitals here in our province have not conducted the formal training of nurses for too long, and this brings about so many challenges in our profession, leading to nurses not having the knowledge and skills for the work. This poses a challenge to patient care and supervision because some of us do not know what to do.’ (FG3, member 15, female participant)

**Sub-theme 2.2: Lack of training of supervisors:** According to the participants, many supervisors appear to lack sufficient training in supervision, as seen by their apparent inability to know what they were supposed to oversee. They said that because they are unsure of what to oversee, the supervisors are not always eager to keep an eye on them. The following are the direct quotes of participants:

‘The challenge that nursing faces is that there are some professional nurses who are lazy and do not know what to do when they are on duty, and they tend to harass other nurses and patients. It seems they are not sure of what to do when on duty and hide behind a negative attitude. Some of the professional nurses are not well trained, which is why they are unsure of what they are doing, as they lack the skills to supervise us.’ (FG2, member 08, female participant)

‘This profession is challenged by professional nurses who lack professionalism and competency in supervising nursing care, and they end up not supervising; this has led to challenges in the supervision.’ (FG5, member 23, male participant)

Some participants said that some of the supervisors lacked the necessary training, making them incompetent at supervising. The participants also mentioned that a large number of supervisors are not competent.

#### Theme 3: Suggested attributes of a good supervisor

According to the participants, a supervisor must possess specific positive qualities that motivate employees to persevere through difficulties to supervise effectively. The qualities mentioned were crucial for nursing care supervision. The participants cited the following attributes of a good supervisor: approachability, avoidance of favouritism, and possessing strong communication skills.

**Sub-theme 3.1: Approachable and avoid favouritism:** An approachable person is pleasant, upbeat, and accessible. For supervision to be effective, supervisors need to be approachable. It is difficult for nurses and patients to reach supervisors who are unapproachable, which makes supervision difficult. When it comes to nursing care supervision, a friendly supervisor is essential. These are some of the quotes from the participants:

‘The supervisors are our resource people in times of need, and they must have the positive qualities of being personable and courteous. Additionally, they must not exercise favouritism.’ (FG1, member 05, female participant)

‘I see that professional nurses should also treat us the same and not practice favouritism. By this, I see that their supervision of nursing care was best as the task allocation will be the same throughout the same categories of nurses. There will be no one who will not be given tasks that are not the same as others because we will be treated in the same way.’ (FG6, member 32, female participant)

According to the participants, a supervisor needs to be friendly to both staff and patients in order to properly oversee. According to some participants, managers should be approachable and kind to everyone so that employees feel comfortable and can concentrate on their work without any concerns.

Some of the participants indicated that the supervisors should treat them the same and avoid favouritism.

**Sub-theme 3.2: Good communication skills:** Participants indicated that to create an atmosphere favourable to nursing care supervision, a supervisor should be able to communicate with both staff and patients. Other participants highlighted that a supervisor who communicates poorly creates an environment that hinders supervision. The following are the participants’ quotes:

‘The supervisors [*Operational managers*] should have good communication skills so that they can communicate well with us. This will also create an environment of trust wherein we can discuss with them whenever we have any challenge.’ (FG5, member 28, female participant)

‘The supervisor should also improve on the way they speak to us as junior nurses because you will find that the patient has requested a bedpan and the professional nurses respond by saying the once for the bedpan shall come and attend to you or telling the patients that they are assistants and not nurses.’ (FG6, member 35, female participant)

#### Theme 4: Supervisory role and responsibility of professional nurses

It is the supervisor’s responsibility to make sure that nursing tasks are completed on time and accurately. It is crucial for the supervisor to make sure that nursing care is being supervised. Supervisors are required to fulfil certain responsibilities while on duty to enhance employee performance and motivate employees to perform to the best of their abilities. Participants reported that some of the roles supervisors must perform include delegating to staff, providing direct supervision, and rewarding good performance.

**Sub-theme 4.1: Delegation of staff:** Supervisors are responsible for ensuring that the assigned individual can perform the assigned task. Delegation of staff is one of the important activities that ensures patients receive the care required, thus contributing to effective supervision of nursing care. The participants expressed the delegation of staff as the supervisory responsibility of professional nurses. The following are the quotes for the participants:

‘What I see is that the supervision that goes well is when they have delegated us the responsibilities, because when you have been delegated, you know the tasks that you must do for the day, even if there are some tasks that can be added, you know your expectations for the day. Delegation also assists them because some of the important tasks cannot remain unattended.’ (FG3, member 13, female participant)

**Sub-theme 4.2: Direct supervision:** Participants think that efficient nursing care supervision results from direct supervision. Thus, it is imperative that professional nurses are always available to support their subordinates. To overcome the difficulty of supervising nursing care, the registered staff and auxiliary nurses believed that direct supervision improved their work performance. Direct supervision was mentioned as a method to enhance nursing care supervision. The following are the quotes from the participants:

‘Professional nurses must always be present in the ward to check on the work of subordinates. This strategy addresses the challenge of supervision of nursing care. Professional nurses must practice direct supervision of nursing care; without direct supervision, staff members always end up not doing their responsibilities.’ (FG1, member 05, female participant)

‘The professional nurses are also responsible for direct supervision of patient care; therefore, the challenges of supervision will be solved. Direct supervision of nursing care improves staff performance, thus addressing the challenges of supervision of nursing care.’ (FG3, member 13, male participant)

**Sub-theme 4.3: Rewarding good performance:** Employees believed they needed inspiration to complete the task. Motivation can occasionally originate from outside sources, such as rewards for excellent work. Rewarding nurses for their efforts is another way to inspire them to work at their highest level. One tactic mentioned by participants to enhance nursing care supervision is rewarding performance. The following are the participants’ quotations:

‘The managers must really motivate us to do the work to the best of our ability so that there will be improved supervision of nursing care. The managers must also reward good performance to boost staff morale.’ (FG 1, member 04, female participant)

‘The manager must always take care of the workers so that they can render quality patient care. The manager can reward good performance. The staff will become effective, and supervision of nursing care shall improve.’ (FG3, member 15, female participant)

## Discussions

Proctor’s supervision model underpinned the discussion of the findings of this study, and the experiences of participants were discussed based on the model. The supervision of nursing care is adversely affected by increased workload, as inefficient work results when there is excessive labour and not enough hands, lowering the standard of nursing care. Proctor’s supervision model states that effective supervision leads to greater nurse efficiency; however, due to a lack of staff, increased workload may lead to greater job dissatisfaction. The quality of nursing care is compromised by increased workload, resulting in patients’ complaints, as participants occasionally attend to patients’ basic needs while neglecting other duties.

According to the findings of a study conducted in Manila, Philippines, by Bautista et al. (2020), high workload was identified as the most common stressor, as it may lead to a lack of job satisfaction. According to a study conducted in Accra, Ghana, by Achempim-Ansong et al. (2021), increased workload results in poor task performance, whereas Proctor’s supervision model highlights that the formative component enables nurses to improve their knowledge and practices, which positively influences their job performance. A study conducted in Newcastle, United Kingdom, by Rothwell et al. (2021:4) indicated that busy work conditions made it difficult for supervisors to find time for supervision, which ultimately limited their flexibility and quality of supervision when they did find time. Studies conducted outlined that a shortage of staff has a negative influence on the supervision of nursing care because of the high workload on both the supervisors and participants, which compromises the quality of nursing care to be rendered, resulting in poor patient outcomes and increased patient complaints.

Consequently, the international studies’ findings are also relevant in the South African supervision context, as supervision is severely affected by a shortage of staff and resources in some hospitals, even though in some hospitals, it is not like that, as there is a sufficient number of supervisors and equipment. In some areas, there is a maldistribution of these resources and personnel, which affects the nursing care and supervision.

Absenteeism, according to Khateeb (2024), is when an employee fails to report for work during a designated time. Absenteeism is a challenge to the supervision of nursing care and is also negatively impacting the quality of nursing care because of the constant shortage of staff due to absenteeism. In a study conducted in Pakistan by Pervez, Kousar and Asghar (2023), participants agreed that patient care and organisational productivity are directly affected by nurse absenteeism, which is linked to challenges in the supervision of nursing care.

Staffing shortages have made fatigue and burnout syndrome extremely prevalent among nurses. Studies conducted reported that absenteeism and staff shortages are the main causes of exhaustion and burnout syndrome in the nursing profession. According to a study conducted in London, United Kingdom, by Razai, Kooner and Majeed (2023), several things can lead to burnout, such as an increase in workload, a lack of support, a lack of autonomy and control, a stressful work environment, and moral harm from not being able to satisfy patients’ needs and demands. Achempim-Ansong et al. (2021) agreed with participants that being supervised and experiencing exhaustion are challenges; however, Proctor’s supervision model highlighted increased self-esteem among participants when they are effectively supervised, but this self-esteem is challenged due to burnout and fatigue caused by a shortage of staff. However, the quality of nursing care and productivity are compromised as a result.

For an organisation to achieve a high productivity level, staff members must receive training, either formally or informally; likewise, it is necessary for hospitals to improve service delivery. Proctor’s restorative dimension of the supervision model supports professional and skills development among nursing staff, which can be enhanced through effective supervision. Proctors’ formative dimension of the supervision model further indicates that once staff members are trained, they become knowledgeable and competent and are able to adhere to nursing standards, thereby promoting quality and clinical audits; hence, the need for effective supervision (normative dimension). While management conducts training for personnel, the staff are responsible for attending training as scheduled. A few individuals mentioned the difficulty of not having enough training opportunities, which is causing them to stagnate rather than advance. Additionally, they stated that hospitals no longer offer in-service training, depriving them of the opportunity to receive the training. According to a study conducted in South Africa, in Limpopo, by Nyelisani, Makhado and Luhalima (2023), continuous development assists nurses in becoming knowledgeable and skilled in their profession, leading to enhanced professional growth. This means that staff nurses and auxiliary nurses are empowered with the knowledge and skills to deliver high-quality nursing care.

Lack of supervisory competence was identified in a number of studies as a barrier to effective supervision. Rothwell et al. (2021) supported the participants, indicating that a lack of training for supervisors was a barrier and resulted in ineffective supervision. Additionally, they claimed that supervisors’ lack of knowledge of professional guidelines (i.e. standards established by regulators), their role and responsibilities as supervisors, ethical standards established by employers, and insufficient educational preparation resulted in substandard supervision. Consequently, the international study findings are also applicable to the South African context, as insufficient educational preparation of supervisors, which is evidenced by a lack of competence and skills, results in challenges in the supervision of nursing care.

Effective communication skills were reported in several studies as essential for supervisors to interact with staff members and enhance nursing care supervision. To improve nursing care supervision, some studies stated that supervisors should be proficient communicators who can interact with both staff and patients. The study conducted in Palestine by Sharkiya (2023) found that participants agreed that better patient care outcomes are mostly dependent on communication. Improved results and better health outcomes are also linked to effective communication, as it increases compliance, helps manage emotions, and improves patient satisfaction. Therefore, supervisors should have good communication skills to interact with all members of the disciplinary team, including the patients.

Proctors’ normative dimension of the supervision model highlights adherence to ethical standards, with delegation of staff and direct supervision as core practices that need to be followed to improve supervision. Several studies have reported that staff delegation is one of the practices that enhance employee performance, ensuring that everyone is focused on the work and every task is completed on time. According to Jooste (2025), delegation is the process of assigning responsibility for carrying out a task to someone else. However, staff delegation is essential for supervision to ensure that there is timely task completion and high-quality patient care is rendered. According to a study conducted in Australia by Walker et al. (2021), supervision and delegation are critical leadership abilities for supervisors in providing quality nursing care, thereby improving nursing care supervision.

Direct supervision, according to the SANC’s position statement (SANC 2021), occurs when an experienced professional nurse provides supervisees with direction. Effective and consistent delegation of power is essential to enhancing good patient care results (Moradi, Rezaei & Alavi 2024). To ensure proper delegation of authority, three key elements, viz., accountability, authority, and responsibility, must be considered. Nurses are responsible for keeping the patients safe, as they have the power and skills to do so, and they are always held accountable for the care they render. Subsequently, the international studies’ findings are also applicable to the South African context of delegation of tasks and direct supervision as a means to improve supervision of nursing care.

One of the things that drives employees to do well at work is rewarding them for their efforts.

Employees should be motivated to reach their full potential, so the manager needs to further inspire them to enhance their productivity. Supervisors are supposed to inspire employees to work diligently so as to improve patient outcomes. In the study conducted in the United States by Waltz et al. (2020), participants agreed that employees who are appreciated through rewards like verbal or written praise experience increased motivation, which can lead to improved patient care.

### Limitations

The findings of this study have revealed that staff nurses and auxiliary nurses should work under the supervision of registered professional nurses, and consequently, quality nursing care can be delivered. The limitation of the study was that it was conducted in three selected regional hospitals; therefore, the findings cannot be generalised to other hospitals and other provinces. Additionally, the study focused on one of South Africa’s nine provinces; its applicability to other provinces may be limited.

### Recommendations

The researcher determined the experiences of staff nurses and auxiliary nurses regarding the supervision of nursing care by professional nurses in the selected regional hospitals of Limpopo province. The study recommends that the shortage of staff should be addressed in order to improve supervision by professional nurses. Furthermore, the management should strengthen the retention of existing nurses in order to retain the very few nurses that are available through the introduction of retention packages, such as rewarding good performance. Debriefing of staff is necessary and may improve job satisfaction and communication; therefore, nursing managers must engage the staff and refer them timeously to debriefing sessions. Mentoring and coaching should be part of the nursing profession in the pursuit of mature and well-developed nurses. The management should address the lack of training of staff through fostering life-long learning to avoid waiting for training by the institution. Nursing education should incorporate the introduction of a supervision module for staff nurses and auxiliary nurses to highlight their expectations during supervision by professional nurses. The study further recommends that there should be a policy for supervision by professional nurses. Future research should be conducted in other provinces and hospitals in order to generalise the findings.

## Conclusion

The study aimed to explore the experiences of staff nurses and auxiliary nurses regarding the supervision of nursing care by professional nurses. The study findings highlighted the experiences of staff nurses and auxiliary nurses regarding the supervision of nursing care by professional nurses. These findings need to be addressed so that the supervision can be improved, which will result in a reduction of patients’ complaints and lawsuits.
